# Employment of 1-Methoxy-5-Ethyl Phenazinium Ethyl Sulfate as a Stable Electron Mediator in Flavin Oxidoreductases-Based Sensors

**DOI:** 10.3390/s20102825

**Published:** 2020-05-15

**Authors:** Maya Fitriana, Noya Loew, Arief Budi Witarto, Kazunori Ikebukuro, Koji Sode, Wakako Tsugawa

**Affiliations:** 1 Department of Biotechnology and Life Science, Graduate School of Engineering, Tokyo University of Agriculture and Technology, 2-24-16 Naka-cho, Koganei, Tokyo 184-8588, Japan; s206376w@st.go.tuat.ac.jp (M.F.); ikebu@cc.tuat.ac.jp (K.I.); 2 Faculty of Biotechnology, Sumbawa University of Technology, Jl. Raya Olat Maras, Batu Alang, Moyo Hulu, Sumbawa Besar 84371, Indonesia; witarto@gmail.com; 3 Joint Department of Biomedical Engineering, The University of North Carolina at Chapel Hill and North Carolina State University, Chapel Hill, NC 27599, USA; noya-loew@rs.tus.ac.jp; 4 Department of Pure and Applied Chemistry, Faculty of Science and Technology, Tokyo University of Science, 2641 Yamazaki, Noda, Chiba 278-8510, Japan

**Keywords:** 1-methoxy-5-ethyl phenazinium ethyl sulfate, disposable enzyme sensor, lactate oxidase, glucose dehydrogenase, fructosyl peptide oxidase, electrochemical enzyme sensor, biomedical engineering

## Abstract

In this paper, a novel electron mediator, 1-methoxy-5-ethyl phenazinium ethyl sulfate (mPES), was introduced as a versatile mediator for disposable enzyme sensor strips, employing representative flavin oxidoreductases, lactate oxidase (LOx), glucose dehydrogenase (GDH), and fructosyl peptide oxidase (FPOx). A disposable lactate enzyme sensor with oxygen insensitive *Aerococcus viridans*-derived engineered LOx (*Av*LOx), with A96L mutant as the enzyme, was constructed. The constructed lactate sensor exhibited a high sensitivity (0.73 ± 0.12 μA/mM) and wide linear range (0–50 mM lactate), showings that mPES functions as an effective mediator for *Av*LOx. Employing mPES as mediator allowed this amperometric lactate sensor to be operated at a relatively low potential of +0.2 V to 0 V vs. Ag/AgCl, thus avoiding interference from uric acid and acetaminophen. The lactate sensors were adequately stable for at least 48 days of storage at 25 °C. These results indicated that mPES can be replaced with 1-methoxy-5-methyl phenazinium methyl sulfate (mPMS), which we previously reported as the best mediator for *Av*LOx-based lactate sensors. Furthermore, this study revealed that mPES can be used as an effective electron mediator for the enzyme sensors employing representative flavin oxidoreductases, GDH-based glucose sensors, and FPOx-based hemoglobin A1c (HbA1c) sensors.

## 1. Introduction

Electrochemical enzyme sensors are the most widely studied form of biosensors due to their simple construction and achievable adequate performance. As is commonly known, in electrochemical enzyme sensors, the substrate (or analyte) is oxidized by a redox enzyme, which results in a product and a reduced cofactor of the enzyme in the reductive half reaction; in other words, electrons are transferred from the substrate to the cofactor of the enzyme [1]. For example, lactate is oxidized by the flavoenzyme lactate oxidase (LOx) harboring flavin mononucleotide (FMN) as a cofactor [2,3,4,5,6]. This results in pyruvate and reduced flavin. In first-generation electrochemical enzyme sensors, the reduced cofactor is reoxidized, and molecular oxygen (O_2_) is reduced to hydrogen peroxide (H_2_O_2_) in the subsequent oxidative half reaction [7,8,9]. The resulting H_2_O_2_ is then oxidized at the surface of the electrode, which is held at an appropriate potential; thus, the electrons are transferred to the electrode and can be measured as an electrochemical current signal. However, the oxidation of H_2_O_2_ requires a high potential (≥+0.6 V vs. Ag/AgCl [1,9]). At this high potential, oxidation of other redox substances in blood can occur, which leads to an erroneous increase in the response current and can cause serious problems [1]. In second-generation electrochemical biosensors, this problem is solved by utilizing an artificial electron mediator to transfer electrons from the reduced cofactor of the enzyme to the electrode. Mediators are preferentially used, especially in disposable strip-type enzyme sensors, because they allow a lower application potential than what is necessary to oxidize H_2_O_2_, which leads to fewer errors due to redox interference.

Second-generation electrochemical biosensors for lactate are currently commercially available for several applications, including self-monitoring [10] and point-of-care testing [11] of blood lactate. Blood lactate levels are a relevant parameter in clinical diagnosis and sports medicine, and the availability of disposable enzyme sensors for the determination of blood lactate is increasing.

LOx derived from *Aerococcus viridans* (*Av*LOx) is widely used in lactate enzyme sensors [12,13,14,15,16,17,18,19], including in commercially available lactate sensor strips [11]. However, LOx utilizes O_2_ as an electron acceptor, which causes inherent problems in second-generation sensors and results in a lower sensor signal. This problem is common to second-generation biosensors utilizing oxidases and can be solved by utilizing dehydrogenases, which do not utilize O_2_ as an electron acceptor. Our group recently engineered *Av*LOx to have a very low reactivity to O_2_, thus converting the enzyme into a dehydrogenase [18]. By utilizing this engineered *Av*LOx, an A96L mutant, the influence of O_2_ is minimized. Thus, utilizing this mutant enzyme can increase the accuracy of lactate sensors.

Attempts are continuing to improve the performance of second-generation lactate sensors, including improving their accuracy and shelf life. In addition to the enzyme, the mediator is a key element in second-generation electrochemical enzyme sensors, so the choice of mediator to be employed in enzyme sensors is one way to improve their performance. When choosing the mediator, the preference of the enzyme for the mediator as its electron acceptor must to be considered, as it affects the efficiency of the electron transfer from the cofactor of the enzyme to the electrode surface and thus determines the suitability of the mediator for sensor construction. Studies have shown that the availability of a mediator may be influenced by electrostatic interactions between the mediator and the enzyme surface or by steric hindrance between the mediator and the enzyme [19,20,21,22,23]. To avoid interference from the oxidation of redox substances present in the sample and thus minimize errors in the response of the biosensor, the biosensor should be operated at a low potential, which can be achieved with a mediator with a low redox potential [24]. Another parameter to be considered in the search for a good mediator is its stability under the sensor fabrication and storage of sensors, where the mediators will be exposed under several conditions, i.e., at room temperature, high humidity, and under the light illumination.

In a recent study, we reported on the mediator preference of *Av*LOx [19]. The light stable form of phenazinium methyl sulfate (PMS), a popular redox dye in spectrometric assays, 1-methoxy-5-methyl phenazinium methyl sulfate (mPMS), was shown to be a more effective mediator for *Av*LOx than potassium ferricyanide [19], a popular mediator commonly used in commercial enzyme sensors. Hexaammine ruthenium(III) chloride, a mediator that is currently gaining attention in the development of enzyme sensors due to its stability, i.e., to light irradiation, and low redox potential (−0.11 V vs. Ag/AgCl [25]) compared to potassium ferricyanide (redox potential +0.23 V vs. Ag/AgCl [26]), was not utilized as an electron acceptor by *Av*LOx [19]. The lactate sensors employing mPMS as the mediator showed a higher sensitivity and a wider linear range than those employing potassium ferricyanide as the mediator. Due to the low redox potential of mPMS (−0.11 V vs. Ag/AgCl [27]), the lactate sensors can be operated at a low potential. Therefore, employing mPMS as the mediator for lactate sensors based on *Av*LOx improves the accuracy of the lactate sensors by avoiding interference from redox substances in the blood [19]. A commercially available blood lactate sensor based on LOx that employs mPMS in combination with hexaammine ruthenium(III) chloride supports these findings [10]. In this commercial lactate sensor, both the effectivity of mPMS as the primary electron acceptor for LOx and the low potential of hexaammine ruthenium(III) chloride are exploited by fabricating lactate sensor strips with a low amount of mPMS to act as the primary electron acceptor and mediator between the enzyme and secondary electron acceptor and a high amount of hexaammine ruthenium(III) chloride to act as the secondary electron acceptor and mediator between the primary electron acceptor and the electrode [10].

Despite its many advantages, however, hexaammine ruthenium(III) chloride contains the rare metal ruthenium, the use of which, considering preservation and sustainability, should be avoided in the future. The organic mediator mPMS is easily synthesized and does not contain any rare elements. Unfortunately, mPMS is not stable in acidic to neutral solutions [27,28,29]. The low stability of mPMS can shorten the shelf life of enzyme sensors employing this mediator.

In 2018, 1-methoxy-5-ethyl phenazinium ethyl sulfate (mPES) became commercially available. This new mediator is stable in solution over a wide pH range [29,30]. Therefore, *Av*LOx-based lactate sensors containing mPES are expected to have a longer shelf life than corresponding sensors containing mPMS. The similar structure and similar electrochemical characteristics of mPES and mPMS suggest that sensors with mPES as the mediator can be developed with negligible interference from redox substances and thus similarly high accuracy. However, the utilization of mPES as a mediator in enzyme sensors has yet to be reported.

In this study, disposable, strip-type enzyme sensors based on representative flavin oxidoreductases, *Av*LOx, *Aspergillus flavus*-glucose dehydrogenase (*Af*GDH), and *Phaeosphaeria nodorum*-fructosyl peptide oxidase (*Pn*FPOx), employing mPES as an electron mediator, were constructed to assess the universal applicability of mPES as a mediator in enzyme sensor strips.

## 2. Materials and Methods

### 2.1. Materials and Apparatus

Sucrose, Tween 20, and silica bead desiccant were purchased from Kanto Chemical Co., Inc. (Tokyo, Japan). Sodium l-lactate was purchased from Sigma-Aldrich (St. Louis, MO, USA). d(+)-Glucose was purchased from Wako Pure Chemical Industries Ltd. (Osaka, Japan). Fructosyl valine was purchased from Santa Cruz Biotech, Inc. (Dallas, TX, USA). l(+)-Ascorbic acid was purchased from Nacalai Tesque, Inc. (Kyoto, Japan). Uric acid and 4′-hydroxyacetanilide were purchased from Tokyo Chemical Industry Co., Ltd. (Tokyo, Japan), while mPES was kindly provided by Dojindo Laboratories (Kumamoto, Japan). All other chemicals were of reagent grade.

Screen-printed carbon electrodes (SPCEs, working electrode [WE]: carbon 2.4 mm^2^; reference electrode [RE]: silver/silver chloride [Ag/AgCl]; counter electrode [CE]: carbon) were kindly supplied by i-SENS (Seoul, Republic of Korea). All electrochemical measurements were carried out using a VersaSTAT4 potentiostat from Princeton Applied Research (Princeton, NJ, USA).

### 2.2. Enzyme Preparation and Activity Evaluation

The *Av*LOx A96L mutant was produced by following the methods of Hiraka et al. [18] with the following minor modifications. The cells of *E. coli* harboring the *Av*LOx A96L mutant were grown in 100 mL medium using 500 mL baffle flasks and incubated at 30 °C and 120 rpm for 36 h. The dye-mediated lactate dehydrogenase activities were measured in 20 mM PPB (pH 7.0) containing 0.06 mM 2,6-dichloroindophenol (DCIP), 4 mM phenazine methosulfate (PMS), and various concentrations of sodium L-lactate. The dehydrogenase activities were determined by monitoring the reduced DCIP at 600 nm based on the molar absorption coefficient of DCIP at pH 7.0 (16.3 mM^−1^ cm^−1^). One unit of dehydrogenase activity was defined as the amount of enzyme necessary to catalyze the reduction of 1 μmol DCIP per minute at 25 °C. These assays were performed in triplicate. Production and activity evaluation of the engineered glucose dehydrogenase V149C/G190C mutant derived from *Aspergillus flavus* (*Af*GDH CC mutant) and fructosyl peptide oxidase N56A mutant derived from *Phaeosphaeria nodorum* (*Pn*FPOx N56A mutant) were carried out according to the methods by Sakai et al. [31] and Kim et al. [32], respectively.

### 2.3. Fabrication of Lactate Sensors and Evaluation of the Sensor Response

**Figure 1:**
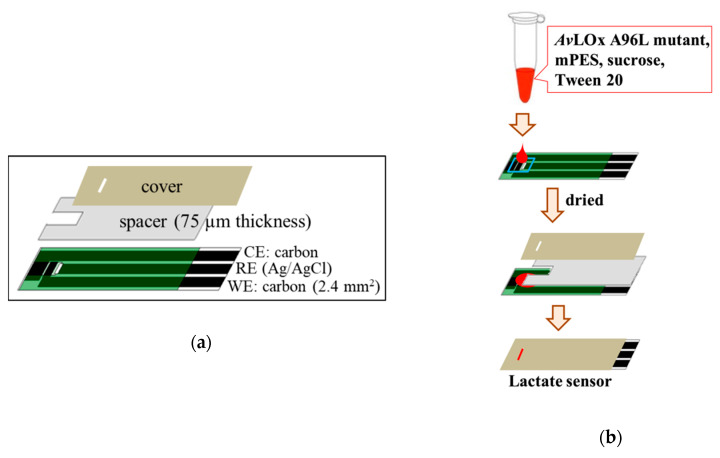
Illustration of screen-printed carbon electrode (SPCE) used in this study; (**a**) components of the SPCE and (**b**) construction of lactate sensor.

A mixture of *Av*LOx, mPES, sucrose, and Tween 20 in 100 mM PPB (pH 7.0) was prepared. One microliter of the mixture, containing 1 U enzyme, 100 mM mediator, 4% sucrose, and 2% Tween 20, was deposited onto the WE of an SPCE. The mixture was then dried onto the SPCE at room temperature (RT) at less than 1% relative humidity (RH) for 3 h. This resulted in an amount of 1 U enzyme, 100 nmol mPES, 40 μg sucrose, and 20 μg Tween 20 per sensor strip. A spacer (75 µm thickness) and a cover were attached to the dry modified SPCEs to form a µL-volume capillary space above the electrodes, resulting in disposable lactate sensors. Figure 1 shows the configuration of SPCE used in this study and the procedure to prepare lactate enzyme sensor. Chronoamperometry (CA) measurements were carried out to evaluate the response currents toward lactate by loading 1.0 µL lactate (0–50 mM) onto the sensor strip, which was connected to the potentiostat. A potential of +0.2 V vs. Ag/AgCl was applied 60 s after the addition of lactate, and the response currents were monitored for another 60 s. The sensitivity, the linear range, the limit of detection (LOD), the limit of quantitation (LOQ), and reproducibility based on the relative standard deviation (RSD) value were calculated as follows.

Sensitivity (µA/mM) = the slope of the calibration curves obtained from plotting lactate concentrations versus response currents.

Linear range (mM) = the range of lactate concentration obtained from the linear line of the calibration curves of lactate concentrations versus response currents.

$$\mathrm{LOD}\;\left(\mathrm{mM}\right)=\frac{3.3\;\times \;\mathrm{standard}\;\mathrm{deviation}\;\mathrm{of}\;\mathrm{background}\;\mathrm{current}\;\left(0\;\mathrm{mM}\;\mathrm{lactate}\right)}{\mathrm{slope}\;\left(\mathrm{sensitivity}\;\mathrm{of}\;\mathrm{lactate}\;\mathrm{sensor}\right)}$$

$$\mathrm{LOQ}\;\left(\mathrm{mM}\right)=\frac{10\;\times \;\mathrm{standard}\;\mathrm{deviation}\;\mathrm{of}\;\mathrm{background}\;\mathrm{current}\;\left(0\;\mathrm{mM}\;\mathrm{lactate}\right)}{\mathrm{slope}\;\left(\mathrm{sensitivity}\;\mathrm{of}\;\mathrm{lactate}\;\mathrm{sensor}\right)}$$

$$\mathrm{RSD}\;\left(\%\right)=\frac{100\;\times \;\mathrm{standard}\;\mathrm{deviation}\;\mathrm{of}\;\mathrm{particular}\;\mathrm{point}\;\left(i.e.,\;\mathrm{for}\;\mathrm{currents}\;\mathrm{obtained}\;\mathrm{with}\;2\;\mathrm{mM}\;\mathrm{lactate}\right)}{\mathrm{mean}\;\mathrm{of}\;\mathrm{response}\;\mathrm{current}\;\mathrm{with}\;2\;\mathrm{mM}\;\mathrm{lactate}\;\left(N\;=3\right)}100\%$$

### 2.4. Evaluation of Response Currents of Lactate Sensors with Different Application Potentials

Lactate sensors were fabricated according to the method described in Section 2.3. The response currents were evaluated by applying a potential of 0 V, +0.05 V, +0.1 V, or +0.2 V vs. Ag/AgCl.

### 2.5. Evaluation of Storage Stability of Lactate Sensors

Lactate sensors were fabricated by following the method described in Section 2.3 but with 5 U enzyme per strip. The lactate sensors were divided into several sets, containing a minimum of 18 sensors each. Each set was wrapped in aluminum foil. All sets were put in a dark plastic box containing silica bead desiccant and stored at 25 °C for up to 50 days. After 2 days (prestorage), the response currents of the first set of lactate sensors were evaluated with 0–50 mM lactate, and the obtained data set was assigned as a control (Day 0). Subsequent sets of lactate sensors were evaluated after 12, 28, and 48 more days of storage (Day 12, Day 28, and Day 48).

### 2.6. Evaluation of Lactate Sensors toward Interferences

Lactate sensors were fabricated according to the method described in Section 2.3. The sensors were evaluated using mixtures of 2 mM or 10 mM lactate containing 0.17 mM ascorbic acid, 0.1 mM uric acid, or 0.3 mM acetaminophen. As with pure lactate solutions, 1.0 μL of the mixture was loaded onto the lactate sensor, and the response current was monitored.

### 2.7. Employing mPES as an Electron Mediator for Other Enzyme Sensors

The corresponding enzyme sensors were fabricated utilizing the enzymes *Af*GDH or *Pn*FPOx (0.1 U per sensor strip) according to the same method described in Section 2.3. The response currents were evaluated by loading solutions of 0–50 mM glucose or fructosyl valine onto the respective sensor strip based on *Af*GDH or *Pn*FPOx, respectively, and potential was applied 90 s after loading the solution.

## 3. Results

### 3.1. Construction of a Disposable Lactate Sensor

In this study, the versatility of mPES was demonstrated by constructing a disposable type lactate enzyme sensor strip, employing SCPE as the representative disposable electrode, widely utilized in the commercially available glucose enzyme sensor strips. A solution containing mediator and enzyme was dried onto a film electrode, and a spacer and cover were attached to complete the disposable lactate sensor. The novel, stable mPES [29] was employed as the mediator, and O_2_ insensitive *Av*LOx A96L mutant was employed as the enzyme [18]. When the sample lactate solution was loaded into the sensor strip, the LOx and the mediator dissolved, and the enzyme reaction began. The time between the start of the enzyme reaction and application of the potential (start of the electrochemical reaction) is known as the waiting time. The waiting time was optimized to 60 s to achieve complete oxidation of lactate by the enzyme and thus full turnover. The response currents observed after application of the potential were generated by oxidation of the reduced mediator at the electrode; the initial amount of the reduced mediator was proportional to the initial amount of lactate in the sample.

**Figure 2:**
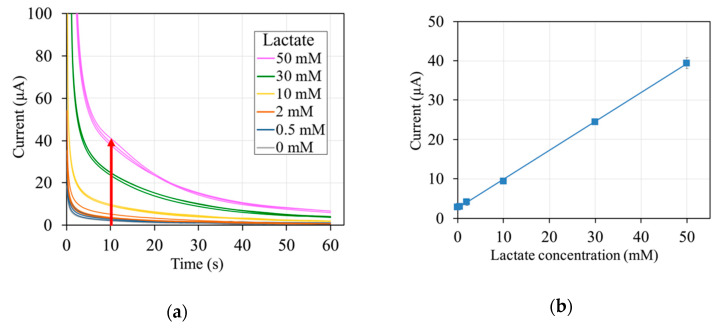
Response currents of lactate sensors; (**a**) time courses and (**b**) calibration curve (linear regression: y = 0.73x + 2.5; R^2^ > 0.99). The arrow in (**a**) indicates increasing lactate concentration. Sensor composition: 1 U *Av*LOx A96L, 100 nmol mPES, 40 μg sucrose, and 20 μg Tween 20. Waiting time: 60 s. Applied potential: +0.2 V vs. Ag/AgCl. N = 3. Sampling point for the calibration curve: at 10 s.

The response currents toward 0–50 mM lactate were monitored amperometrically at an operation potential of +0.2 V vs. Ag/AgCl (operation potential was determined from cyclic voltammograms of the mediator, Supplementary Material Figure S1). An initial high response current was observed, which gradually decreased with time until it reached a steady state. In Figure 2a, the time courses of the response currents, or amperograms, are shown. The current at 10 s after potential application was plotted against the lactate concentrations to obtain the calibration curve (Figure 2b). The observed response currents increased linearly depending on the lactate concentration. A wide linear range of up to 50 mM lactate was obtained. The sensitivity of the lactate sensor, defined as the slope of the linear range, was 0.73 ± 0.12 μA/mM. The lactate sensor also showed good reproducibility at each tested lactate concentration (RSD < 7%) (Table 1). The LOD and LOQ was 0.5 mM and 1.8 mM, respectively. These results show that the lactate sensor was constructed successfully and that lactate can be measured with this lactate sensor.

### 3.2. Optimization of Operation Potentials

**Figure 3:**
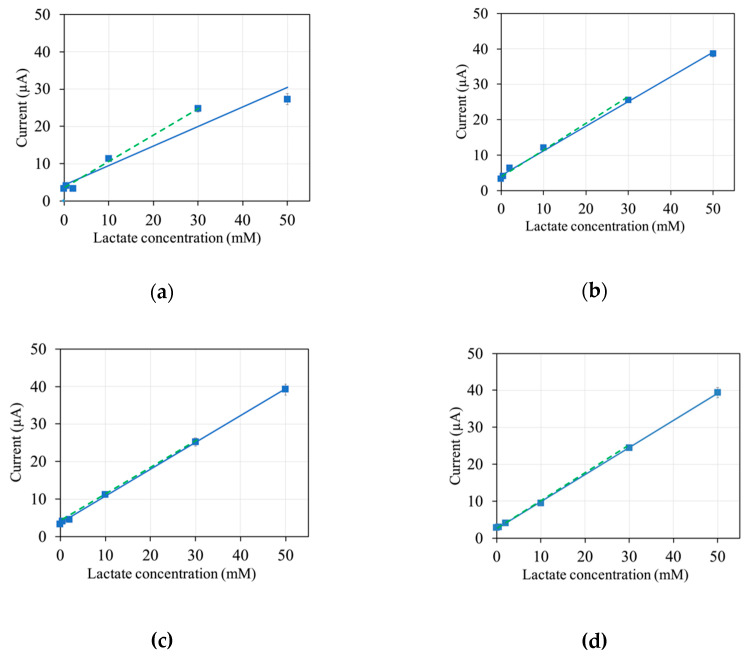
Response currents at different application potentials: (**a**) 0 V, (**b**) +0.05 V, (**c**) +0.1 V, and (**d**) +0.2 V vs. Ag/AgCl. Blue continuous line: linear regression for the range of up to 50 mM; green dashed line: linear regression for the range of up to 30 mM. Sensitivity for the range up to 50 mM: 0.52 µA/mM (R^2^ = 0.93) in (**a**) and 0.69–0.73 µA/mM (R^2^ > 0.99) in (**b**–**d**). Sensitivity for the range up to 30 mM: 0.69–0.73 µA/mM (R^2^ > 0.99) in (**a**–**d**). Sensor composition: 1 U *Av*LOx A96L, 100 nmol mPES, 40 μg sucrose, and 20 μg Tween 20. Waiting time: 60 s. N = 3. Calibration curves at sampling point: 10 s.

To optimize the operation potential, response currents were evaluated with different applied potentials, from 0 to +0.2 V (vs. Ag/AgCl) (Figure 3). A clear dependency of the response current on the lactate concentration was observed at all potentials. However, at 0 V applied, the linear range extended only up to 30 mM (Figure 3a). At higher operating potentials, the linear range extended up to 50 mM (Figure 3b–d). This is evidenced in the fact that the linear regression of the response currents obtained has the same slope and a high R^2^ value with and without the response current value obtained with 50 mM lactate included when a potential higher than 0 V was applied (continuous vs. dashed lines in Figure 3b–d). When 0 V was applied, the response current value obtained with 50 mM lactate significantly deviated from the linear regression line obtained with response current values up to 30 mM lactate (Figure 3a).

A linear range of up to 30 mM lactate, which was obtained at all tested operation potentials, is more than enough to cover the standard range of blood lactate levels in humans, which can increase to a maximum of 25 mM [22]. The lactate sensor showed a good sensitivity of approximately 0.7 μA/mM, defined as the slope of the linear calibration, for the range of up to 30 mM lactate for all evaluated potentials, indicating that the sensor can be used at any operating potential between 0 V and +0.2 V vs. Ag/AgCl for measurements of up to 30 mM lactate. If higher lactate concentrations are expected, an operation potential of +0.05 V or higher should be used.

### 3.3. Interference of Common Redox Substances

**Figure 4:**
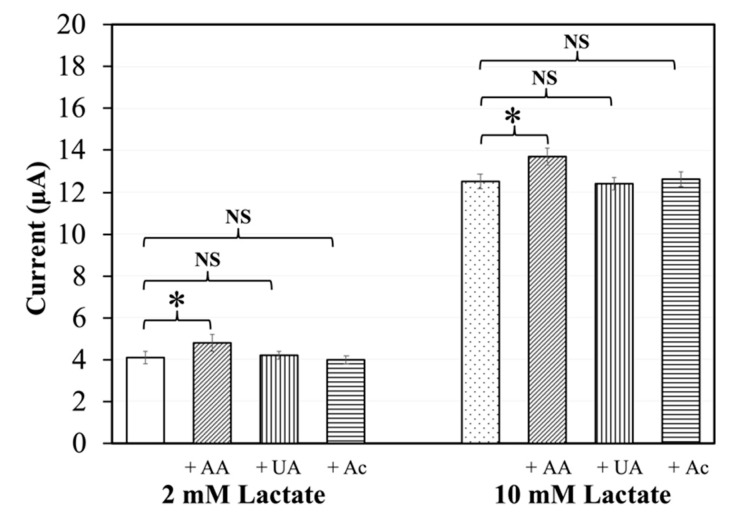
Response currents of lactate sensors to 2 mM or 10 mM lactate in the absence and presence of redox substances: 0.17 mM ascorbic acid (AA), 0.1 mM uric acid (UA) or 0.3 mM acetaminophen (Ac). * Student’s unpaired *t-*test value *p* < 0.05 (suggesting a significant difference between data); NS: *p* > 0.05 (suggesting no significant differences between data). Sensor composition: 1 U *Av*LOx A96L, 100 nmol mPES, 40 μg sucrose, and 20 μg Tween 20. Waiting time: 60 s. Applied potential: +0.2 V vs. Ag/AgCl. N = 3. Sampling point: 10 s.

The response of the lactate sensors in the presence of possibly interfering redox substances was evaluated. For this, mixtures of 2 mM and 10 mM lactate with either ascorbic acid, uric acid, or acetaminophen were loaded into the sensor strips. In the presence of ascorbic acid, the observed response currents were higher than the corresponding signals of samples without ascorbic acid (Figure 4). Student’s unpaired *t*-test showed a *p* value of *<* 0.05, indicating a significant difference. In the presence of uric acid or acetaminophen, there was no significant difference in the response currents compared to corresponding currents in the absence of redox substances (*p >* 0.05). Measurements of samples with 0 mM lactate and 10 mM interferent were also carried out, with corresponding results (Supplementary Material Figure S2). These results suggest that the lactate sensors are affected by ascorbic acid but not by uric acid or acetaminophen.

### 3.4. Storage Stability of the Lactate Sensors

**Figure 5:**
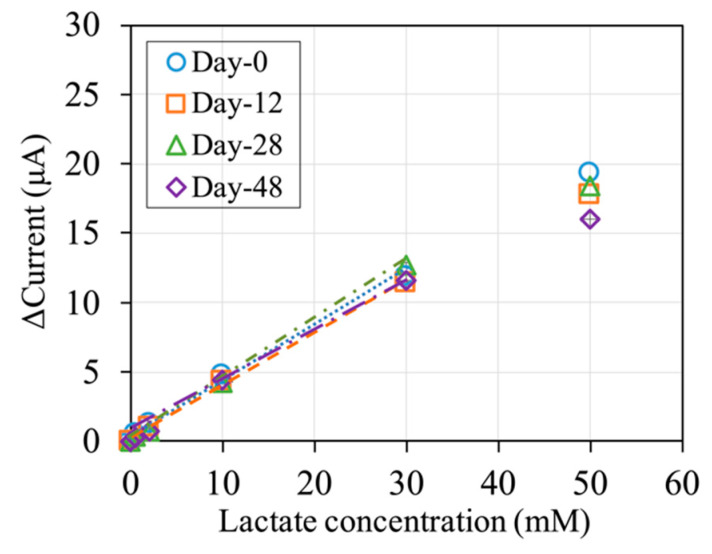
Calibration curves of lactate sensors stored at 25 °C in the dark, evaluated after storage for 2 days (prestorage) + 0, 12, 28, or 48 days. Linear regression was determined for up to 30 mM lactate; sensitivities were 0.38–0.42 µA/mM (R^2^ > 0.99) for all calibration curves. Sensor composition: 5 U *Av*LOx A96L, 100 nmol mPES, 40 μg sucrose, and 20 μg Tween 20. Waiting time: 60 s. Applied potential: +0.2 V vs. Ag/AgCl. N = 3. Sampling point: 10 s.

The storage stability of the lactate sensors was evaluated with sensor strips with 5 U of *Av*LOx A96L per sensor, to avoid the case that enzyme inactivation would be the major factor to show a decrease in the sensor performance. The stability of the sensor with 1 U of *Av*LOx A96L per sensor is shown in Supplementary Material Figure S3, where no significant difference was observed from those of the sensor with 5 U of enzyme. The lactate sensors were stored for up to 2 + 48 days at 25 °C in the dark, with the first 2 days designated as prestorage. Sets of sensors were evaluated at 0, 12, 28, and 48 days of main storage (Figure 5). The sensitivity for calibration of up to 30 mM lactate was approximately 0.4 μA/mM at 0 days of main storage. The lower sensitivity compared to the sensors in Section 3.1 and Section 3.2 can be explained by the increased amount of protein. However, the sensitivity is still acceptably high. The detection limit of the sensor also did not change during the storage for 48 days at 25 °C (Supplementary Material Table S1).

The sensitivity for calibration of up to 30 mM lactate did not change significantly for 48 days of storage (Figure 5). The response current obtained with 50 mM lactate, however, decreased slightly with storage time. To support these findings, electrode strips containing only mediator were prepared and evaluated using a mixture of *Av*LOx A96L mutant and lactate (Supplementary Material Figure S4). The excellent storage stability of the mediator electrode strips at 25 °C and good storage stability at 45 °C suggests that the slight decrease in sensitivity to 50 mM lactate might be due to the enzyme. Therefore, this sensor can be used for measurements of up to 30 mM lactate after up to 48 days of storage at 25 °C in the dark without loss of sensitivity. For measurements of higher lactate concentrations, new lactate sensors should be used.

### 3.5. mPES as an Electron Mediator for Other Enzyme Sensors

**Figure 6:**
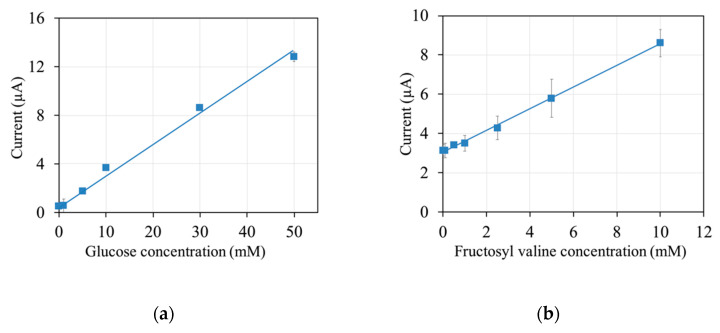
Calibration curves of biosensors utilizing mPES and (**a**) *Af*GDH CC mutant (linear regression: y = 0.25x + 0.65; R^2^ = 0.99) or (**b**) *Pn*FPOx N56A mutant (linear regression: y = 0.55x + 3; R^2^ > 0.99). Sensor composition: 0.1 U enzyme, 100 nmol mPES, 40 μg sucrose, and 20 μg Tween 20. Waiting time: 90 s. Applied potential: +0.2 V vs. Ag/AgCl. N = 3. Sampling point: 10 s.

Finally, to investigate the usability of mPES as a mediator for other enzymes, enzyme sensor strips were constructed utilizing mPES and the diagnostic enzymes *Af*GDH and *Pn*FPOx. The responses of the sensors based on *Af*GDH and *Pn*FPOx toward glucose and fructosyl valine, respectively, were evaluated. The response currents at 10 s were plotted against the analyte concentration, and calibration curves were obtained (Figure 6).

A clear linear dependency of the response currents on the analyte concentration was observed, with a linear range of up to 50 mM glucose for the sensor strips containing *Af*GDH and up to 10 mM fructosyl valine for the sensor strips containing *Pn*FPOx. These results show that both *Af*GDH- and *Pn*FPOx-based enzyme sensor strips were constructed successfully utilizing mPES as a mediator.

## 4. Discussion

In this study, a novel electron mediator, mPES, was introduced as a versatile mediator for disposable enzyme sensor strips, employing representative flavin oxidoreductases, *Av*LOx, *Af*GDH, and *Pn*FPOx.

In our previous work, an oxygen insensitive mutant, *Av*LOx A96L, was constructed, and it was demonstrated the employment of this mutant was the solution to overcome the inherent problem of oxidase that the sensor signal is affected by oxygen [18]. However, we did not conclude the appropriate mediator for the lactate sensor employing *Av*LOx A96L. In the other achievement, we reported the investigation of electron mediators for enzyme sensors employing *Av*LOx, where we concluded that mPMS was the best [19]. However, mPMS is recognized as an unstable mediator, especially in neutral condition [27,28,29]. Therefore, we have been investigating an alternate mediator for LOx-based enzyme sensors, and we came across the use of mPES, which is the derivative of mPMS, but is more chemically stable than mPMS.

The properties of the sensor strips investigated in this study are summarized in Table 1. All three sensors have a linear range that sufficiently covers the range needed for the measurement of blood lactate levels, blood glucose levels, and HbA1c values. All three sensors also have good sensitivity, a low LOD value, a high R^2^ value for the linear regression, and a low RSD of the measurements. These parameters indicate the high accuracy of the sensors. In this study, to demonstrate the versatility of mPES, a SCPE was employed as the representative disposable electrode, which is widely utilized in the commercially available glucose enzyme sensor strips. Current results did not indicate any obstacle issues of mPES to be used for sensors with another electrode material, such as gold, platinum, and palladium, which are conventionally utilized for disposable sensor strips.

The method used in these enzyme sensors based on monitoring of lactate (*Av*LOx A96L), glucose (*Af*GDH), and fructosyl amino acid (*Pn*FPOx) acid is an end-point assay, as has been utilized for commercially available glucose sensors for self-monitoring of blood glucose. Considering the amount of target molecule in 1 µl sample, 0.1 U of enzyme will be theoretically enough to oxidize an entire sample within the setting time for incubation (60 s for lactate and 90 s for glucose and fructosyl amino acid). In these types of enzyme sensors, the enzyme reaction reaches its equilibrium relatively fast, usually before the potential is applied. The response current is an indicator for the concentration of the reduced mediator at equilibrium. For example, in the current experiment with the sample volume of 1 µL with 50 mM of lactate sample, there are 50 nmol of lactate and 1 U of *Av*LOx A96L. Considering the definition of 1 U unit of dehydrogenase activity was defined as the amount of enzyme necessary to catalyze the reduction of 1 μmol (1000 nmol) DCIP in 60 s at 25 °C, which corresponds to the oxidation of 500 nmol of lactate in 60 s, excess amount of *Av*LOx A96L existed on the electrode in this experiment. Usually, enzyme sensor strips contain an excess amount of enzyme, considering the shelf life of a sensor, as the enzyme gradually inactivates during the preservation. We chose 1 U or 5 U of *Av*LOx A96L considering further investigation of mediator stability during the preservation, where enzyme stability would not be the limiting steps. For the investigation of the availability of mPES for *Af*GDH and *Pn*FPOx, where we did not plan a stability investigation, 0.1 U of enzymes were employed with 90 s for enzyme reaction, which provided enough time to arrive at end points for each experiment.

In addition to the characteristics of the enzyme, the characteristics of the mediator are a key factor in determining the properties of second-generation electrochemical biosensors. A commonly considered characteristic of the mediator is its redox potential, which determines the possible operating potentials of the sensor and thus influences the accuracy in terms of sensitivity to redox substance-type interference. Here, mPES has a low redox potential, which results in a low operating potential of +0.2 V. An even lower operating potential can be used with mPES as a mediator, although this might come at the cost of a narrower linear range (Figure 3).

A low operating potential generally means few interferences by redox substances that can be directly oxidized at the electrode. Three common redox substances in blood are ascorbic acid, uric acid, and acetaminophen, with the redox potential approximately +0.16 V, +0.45 V [33], and +0.2 V [34] (vs Ag/AgCl), respectively. In interference tests using the *Av*LOx-based sensors with 2 mM and 10 mM lactate, which are within and above the physiological range of lactate concentration in blood, respectively, we could show that the operating potential of +0.2 V is sufficiently low to eliminate interference by uric acid and acetaminophen (Figure 4). This was further supported by the fact that the sensors showed a response similar to the background (0 mM lactate) to 10 mM uric acid or acetaminophen (Supplementary Material Figure S2a).

However, a small, yet significant, bias in the response current is observed in the presence of ascorbic acid at an operating potential of +0.2 V (Figure 4), despite the potential being lower than the redox potential of ascorbic acid. Furthermore, the response of the sensor strips to 10 mM ascorbic acid was almost as high as that to 10 mM lactate (Figure S2a). Moreover, the response current did not decrease much at lower operating potentials, although a large decrease in the direct oxidation of a substance with the redox potential of ascorbic acid would be expected (Figure S2b). Reports have shown that ascorbic acid interferes by reducing the mediator rather than by being oxidized at the electrode [35,36,37,38,39], as exploited by a commercially available portable analyzer measuring reducing compounds, mainly ascorbic acid, by their ability to reduce the mediator, which is then quantified by oxidizing the mediator at the electrode [40]. Therefore, it is feasible that ascorbic acid can reduce mPES, which is then oxidized at the electrode and thus leads to a false signal.

Another characteristic of the mediator to be considered is its stability. This is especially the case in disposable sensor strips that operate with the end-point principle, meaning that the enzyme reaction is completed before the electrode reaction begins. In contrast to the kinetic principle, in which the mediator is recycled between the enzyme and electrode, for the end-point principle, a large amount of mediator is needed, as no recycling occurs. Thus, the storage stability of the sensor strips relies significantly on the storage stability of the mediator in the strips. Reports show that mPES is more stable than similar mediators, such as mPMS, over a wide range of pH values [29,30]. At highly alkaline conditions, decomposition of mPES due to ring-opening may occur [30]. Generally, neutral pH values are maintained during the fabrication and use of enzyme sensor strips, so that instability due to extreme pH conditions is not of concern. In this study, we investigated the storage stability of *Av*LOx-based sensor strips containing mPES. For this investigation, sensor strips containing more enzyme were used to avoid a decrease in the response current due to enzyme degradation, which led to a lower initial sensitivity due to the higher amount of protein. No significant decrease in the sensitivity in the range of 0–30 mM lactate was observed after 48 days of storage at 25 °C (Figure 5). The mediator itself proved to be stable at temperatures of at least 45 °C (Figure S4). Therefore, employing mPES as a mediator should be a successful step toward the development of sensor strips with an extended shelf life.

These results indicated that mPES is a promising organic mediator for strip-type disposable second-generation electrochemical biosensors, thanks to the superior characteristics of mPES, that is, the low redox potential, compatibility with various diagnostically relevant enzymes, and especially the high stability. The most common mediator, potassium ferricyanide, has a high redox potential (+0.23 V vs. Ag/AgCl [26]). Therefore, the amperometric enzyme sensor with potassium ferricyanide should be operated at high potential, otherwise consequently the signal will suffer from the oxidation current of electrochemically active ingredient potentially existing in samples. Potassium ferricyanide is also a labile molecule, which is easily inactivated during the preservation, especially under light exposure. Hexaammine ruthenium(III) has similar redox potential (−0.11 V vs. Ag/AgCl [25]) to mPES, however, hexaammine ruthenium(III) is not available as the mediator for native *Av*LOx and *Af*GDH as reported [19,20]. In addition, the use of the rare metal element, ruthenium, which has limited existence in the earth, is not recommended as the mediator for a disposable sensor. These inherent issues of using potassium ferricyanide or hexaammine ruthenium(III) as mediators will be overcome by substituting with mPES. PMS and mPMS have been widely utilized as the mediators of a variety of enzyme electrochemical investigations, thanks to their low redox potential (−0.11 V vs. Ag/AgCl [27]). Considering that PMS is a labile molecule toward light exposure, mPMS was developed, however mPMS is not stable at neutral to alkaline condition. mPES keeps low redox potential (−0.14 V vs. Ag/AgCl, Supplementary Material Figure S1) stability under light exposure, but with improved stability over a wide pH range. This study demonstrated that mPES can be used as the electron mediator of a variety of oxidoreductases, and thus, it is the most promising mediator for further studies of electrochemical sensors.

## 5. Conclusions

In this study, a novel electron mediator, mPES, was introduced for disposable enzyme sensor strips, employing representative flavin oxidoreductases, *Av*LOx, *Af*GDH, and *Pn*FPOx, by demonstrating versatility of this mediator including applying potential for the amperometric enzyme sensors, impact of potential redox active ingredients, storage stability, and availability as the electron acceptor for various enzymes. The successfully constructed lactate sensor had a high sensitivity (0.73 ± 0.12 μA/mM) and a wide linear range (0–50 mM lactate). Thanks to the redox potential of mPES, the sensor was operated by applying a low operation potential of +0.2 V vs. Ag/AgCl, or even at low as low as 0 V, without significant loss in sensitivity, and consequently, no interference was observed in the presence of uric acid and acetaminophen. Ascorbic acid reacts with mPES, and thus, precautions need to be implemented to account for ascorbic acid in the sample. Furthermore, the lactate sensors were stable for at least 48 days of storage at 25 °C in the dark, which is unusually long for an organic mediator without the addition of stabilizing agents. The compatibility of mPES with other representative diagnostic enzymes, such as GDH and FPOx, was also approved. Thus, compared to potassium ferricyanide, or hexaammine ruthenium(III) chloride, mPES might be a good alternative mediator to consider for future developments of second-generation electrochemical enzyme sensor strips.

## Figures and Tables

**Table 1:** Properties of biosensors employing 1-methoxy-5-ethyl phenazinium ethyl sulfate (mPES) in this study.

Enzyme	Amount of Enzyme (U/strip)	Linear Range (0 mM to)	Sensitivity (µA/mM)	R^2^	LOD (mM)	RSD (%)
*Av*LOx A96L mutant	1	50 mM ^a^	0.73 ± 0.12	>0.99	0.5	<7
*Af*GDH CC mutant	0.1	50 mM ^b^	0.25	>0.99	2.6	<3 ^d^
*Pn*FPOx N56A mutant	0.1	10 mM ^c^	0.55	>0.99	1.1	<8 ^e^

Analyte: ^a^ lactate, ^b^ glucose, and ^c^ fructosyl valine. LOD: limit of detection; RSD: relative standard deviation. ^d^ for 10 mM, ^e^ for 0.5 and 10 mM.

## Supplementary Materials

The following are available online at https://www.mdpi.com/1424-8220/20/10/2825/s1.
