# Supplementary material for: Employment of 1-Methoxy-5-Ethyl Phenazinium Ethyl Sulfate as a Stable Electron Mediator in Flavin Oxidoreductases-Based Sensors
Source: Sensors (Basel). 2020 May 15;20(10):2825. doi: 10.3390/s20102825 (PMC7284575; doi:10.3390/s20102825)
Supplement: Supplementary file 1 [file sensors-20-02825-s001.pdf]

Supplementary materials

Employment of 1-Methoxy-5-Ethyl Phenazinium Ethyl Sulfate as a Stable Electron Mediator in Flavin Oxidoreductases-Based Sensors

Maya Fitriana [1,2], Noya Loew [3,4], Arief Budi Witarto [2], Kazunori Ikebukuro [1], Koji Sode [3,*] and Wakako Tsugawa [1,*]

1 Department of Biotechnology and Life Science, Graduate School of Engineering, Tokyo University of Agriculture and Technology, 2-24-16 Naka-cho, Koganei, Tokyo 184-8588, Japan; s206376w@st.go.tuat.ac.jp (M.F.); ikebu@cc.tuat.ac.jp (K.I.); tsugawa@cc.tuat.ac.jp (W.T.)
2 Faculty of Biotechnology, Sumbawa University of Technology, Jl. Raya Olat Maras, Batu Alang, 84371 Sumbawa Besar, Indonesia; witarto@gmail.com (A.B.W.)
3 Joint Department of Biomedical Engineering, The University of North Carolina at Chapel Hill and North Carolina State University, Chapel Hill, NC 27599, USA; ksode@email.unc.edu (K.S.)
4 Department of Pure and Applied Chemistry, Faculty of Science and Technology, Tokyo University of Science, 2641 Yamazaki, Noda, Chiba 278-8510, Japan; noya-loew@rs.tus.ac.jp (N.L)
* Correspondence: ksode@email.unc.edu (K.S.); tsugawa@cc.tuat.ac.jp (W.T.); Tel.: +1-919-966-3550 (K.S.); +81- 42-388-7027 (W.T.)

Figure S1

Cyclic voltammograms of mediators

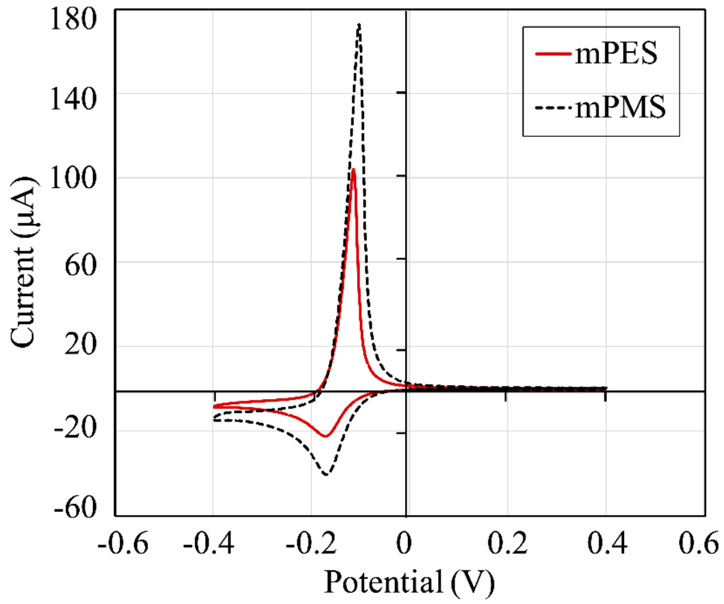

Cyclic voltammetry (CV) measurements were carried out for 1 mM mPMS (black dashed line) or mPES (red solid line) in 100 mM PPB (pH 7.0). Potential range: 0.4 to -0.4 V vs Ag/AgCl; scan rate: 0.1 V/s. Working electrode (WE): glassy carbon (GC); reference electrode (RE): Ag/AgCl; counter electrode (CE): platinum wire.

Oxidation and reduction peaks were observed at -0.11 V and -0.17 V vs Ag/AgCl, respectively, for mPMS and at -0.11 V and -0.18 V vs Ag/AgCl, respectively, for mPES. Thus, the half wave potential of both mediators was -0.14 V vs Ag/AgCl.

Figure S2

Response currents of lactate sensors toward common redox substances

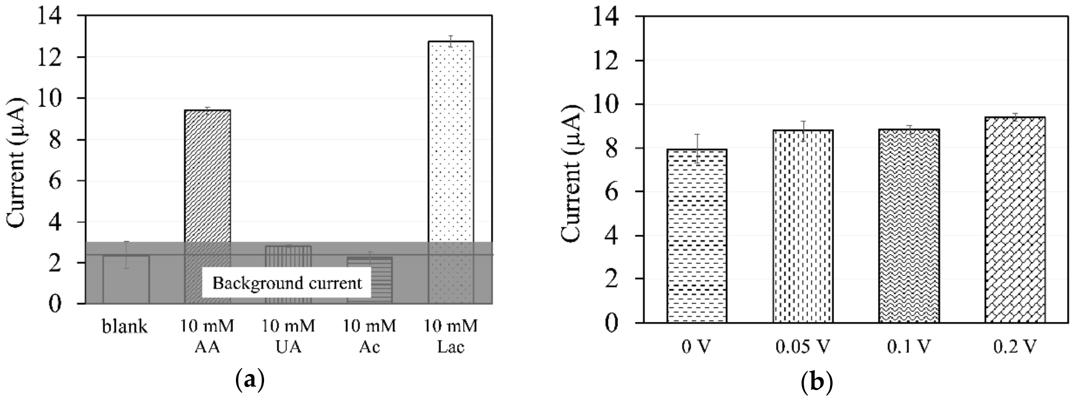

(**a**) Response currents to blank sample and 10 mM ascorbic acid (AA), uric acid (UA), acetaminophen (Ac), and lactate (Lac). Applied potential: +0.2 V vs Ag/AgCl. Gray area: background current. (**b**) Response currents to 10 mM ascorbic acid at various applied potentials.

Sensor composition: 1 U *Av*LOx A96L, 100 nmol mPES, 40 μg sucrose, and 20 μg Tween 20. Waiting time: 60 s. N = 3. Sampling point: 10 s.

Figure S3

Storage stability of lactate sensors with 1 U of *Av*LOx A96L

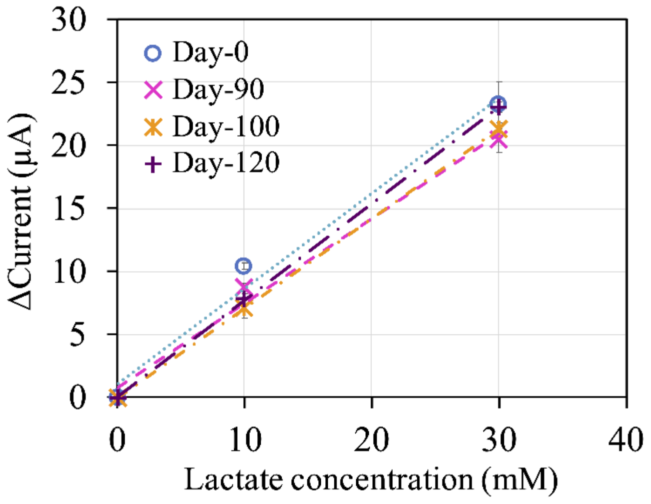

Calibration curves of lactate sensors stored at 25°C in the dark, evaluated after storage for 0, 90, 100 or 120 days. Linear regression was determined for up to 30 mM lactate; sensitivities were 0.68 - 0.77 µA/mM ($R^2 > 0.98$) for all calibration curves. Sensor composition: 1 U *Av*LOx A96L, 100 nmol mPES, 40 µg sucrose, and 20 µg Tween 20. Waiting time: 60 s. Applied potential: +0.2 V vs Ag/AgCl. N = 3. Sampling point: 10 s.

Table S1

The limit of detection (LOD) of the lactate sensor with 5 U *Av*LOx A96L during storage

**Day storage [1]**	0	12	28	48
**LOD (mM)**	1.1	1.1	0.5	1.0

[1] with 2 days prestorage

Figure S4

Stability of mPES electrode strips

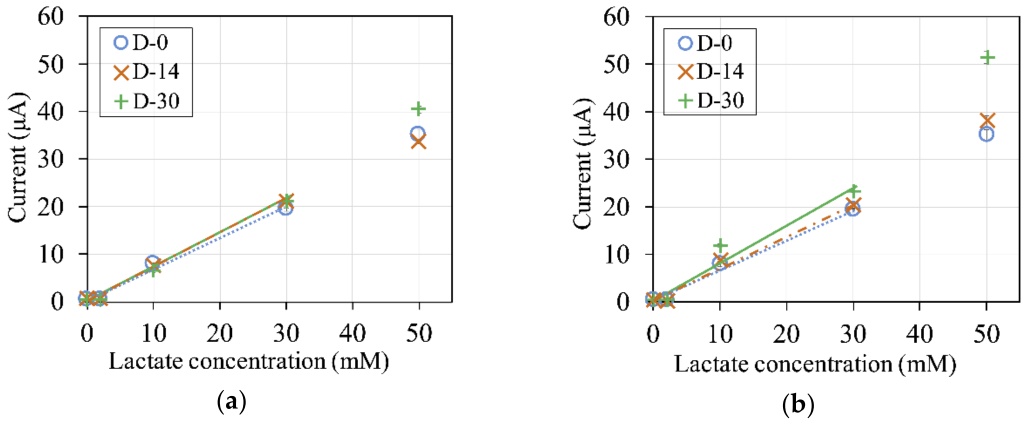

Sensor composition: 100 mmol mPES. Sample composition: 0-50 mM lactate, 5 U/µL *Av*LOx A96L, 4% sucrose, and 2% Tween 20 in 100 mM PPB (pH 7.0). Waiting time: 60 s. Applied potential: +0.2 V vs Ag/AgCl. N = 3. Sampling point: 10 s. Sensor strips were stored for 0, 14, or 30 days at (**a**) 25°C or (**b**) 45°C in the dark. Linear regression: 0-30 mM lactate; sensitivities: 0.65-0.78 µA/mM; $R^2 > 0.95$.
